# Hypertension Medication Recommendation via Synergistic and Selective Modeling of Heterogeneous Medical Entities: Development and Evaluation Study of a New Model

**DOI:** 10.2196/74170

**Published:** 2025-11-25

**Authors:** Ke Zhang, Zhichang Zhang, Yali Liang, Wei Wang, Xia Wang

**Affiliations:** 1 College of Computer Science and Engineering, Northwest Normal University, 967 Anning East Road, Lanzhou, 730070, China, +86 13038769329; 2 Gansu Provincial Hospital, Lanzhou, China

**Keywords:** hypertension medication recommendation, electronic health records, heterogeneous graph, multihead graph attention mechanism, time series selective mechanism

## Abstract

**Background:**

Electronic health records (EHRs) contain comprehensive information regarding diagnoses, clinical procedures, and prescribed medications. This makes them a valuable resource for developing automated hypertension medication recommendation systems. Within this field, existing research has used machine learning approaches, leveraging demographic characteristics and basic clinical indicators, or deep learning techniques, which extract patterns from EHR data, to predict optimal medications or improve the accuracy of recommendations for common antihypertensive medication categories. However, these methodologies have significant limitations. They rarely adequately characterize the synergistic relationships among heterogeneous medical entities, such as the interplay between comorbid conditions, laboratory results, and specific antihypertensive agents. Furthermore, given the chronic and fluctuating nature of hypertension, effective medication recommendations require dynamic adaptation to disease progression over time. However, current approaches either lack rigorous temporal modeling of EHR data or fail to effectively integrate temporal dynamics with interentity relationships, resulting in the generation of recommendations that are not clinically appropriate due to the neglect of these critical factors.

**Objective:**

This study aims to overcome the challenges in existing methods and introduce a novel model for hypertension medication recommendation that leverages the synergy and selectivity of heterogeneous medical entities.

**Methods:**

First, we used patient EHR data to construct both heterogeneous and homogeneous graphs. The interentity synergies were captured using a multihead graph attention mechanism to enhance entity-level representations. Next, a bidirectional temporal selection mechanism calculated selective coefficients between current and historical visit records and aggregated them to form refined visit-level representations. Finally, medication recommendation probabilities were determined based on these comprehensive patient representations.

**Results:**

Experimental evaluations on the real-world datasets Medical Information Mart for Intensive Care (MIMIC)-III v1.4 and MIMIC-IV v2.2 demonstrated that the proposed model achieved Jaccard similarity coefficients of 58.01% and 55.82%, respectively; areas under the curve of precision-recall of 83.56% and 80.69%, respectively; and *F*_1_-scores of 68.95% and 64.83%, respectively, outperforming the baseline models.

**Conclusions:**

The findings indicate the superior efficacy of the introduced model in medication recommendation, highlighting its potential to enhance clinical decision-making in the management of hypertension. The code for the model has been released on GitHub.

## Introduction

### Background

Hypertension represents a prevalent chronic condition and serves as a significant contributor to cardiovascular mortality, making timely pharmacological intervention for blood pressure management crucial [1]. With the increasing trend of an aging population, the growing number of patients with hypertension has placed a significant burden on health care systems [2]. Consequently, automated medication recommendation systems for hypertension have been developed.

### Prior Work and Limitations

Early hypertension medication recommendation methods were primarily rule-based. For example, Wu and Xie [3] developed a hypertension ontology and reasoning rules to recommend appropriate antihypertensive medications to patients. However, these methods relied only on predefined rules and a limited set of case data, neglecting other critical patient information, which resulted in recommendations that lacked flexibility and personalization. In recent years, numerous neural network models for recommending hypertension medications based on electronic health records (EHRs) have been proposed [4,5]. These models have shown improved outcomes and effectively address many limitations inherent in earlier algorithms.

Nevertheless, the complexity of EHR data continues to present significant challenges for medication recommendation tasks, particularly in 2 critical areas: insufficient synergy among heterogeneous medical entities and neglect of temporal dynamics in the patient’s condition.

### Insufficient Synergy Among Heterogeneous Medical Entities

EHR data contains heterogeneous but interrelated medical entities—such as diagnoses, procedures, and medications—that jointly influence treatment outcomes. For example, a diagnosis indicates a patient’s health status, which subsequently informs procedure and medication decisions. Effective modeling of such cross-entity relationships is essential for generating accurate and personalized treatment recommendations.

Existing approaches like Multilevel Medical Embedding (MiME) [6] and graph convolutional transformer (GCT) [7] attempt to model medical concepts and their causal relations using homogeneous graph structures. However, these structures fail to capture the inherent heterogeneity across EHR entities. The Heterogeneous Information Network for Medical Diagnosis (HeteroMed) [8] introduces semantic associations via metapaths, but it does not model dynamic interentity interactions. CausalMed [9] explores causal inferences between treatment elements but overlooks the reinforcement between medications and procedures. Graph transformers of bidirectional encoder representations from transformers on EHRs (GT-BEHRT) [10] integrates graph transformers with temporal modeling yet does not incorporate contextual synergy among entities.

Further, current models inadequately balance efficacy and safety. For instance, Graph-Augmented Memory Networks (GAMENet) [11] uses a drug-drug interaction (DDI) graph to reduce adverse effects but does not consider diagnostic and procedural inputs. Graph-augmented bidirectional encoder representations from transformers (G-BERT) [12] extends to multientity modeling yet omits DDI-aware safety metrics. Competitive neural network (CompNet) [13] faces issues with computational efficiency and model stability on large-scale datasets. Recent work like that by Yang et al [14] (using medication molecular structure graphs) and Li et al [15] (using contrastive learning) enhance representation power, but they still lack a unified mechanism for modeling both “multientity synergy” and “safety constraints.”

### Neglect of Temporal Dynamics in the Patient’s Condition

As a chronic disease, hypertension evolves over time. In clinical practice, treatment strategies are often adjusted based on both current and historical visit data. Hence, capturing the temporal dynamics of the patient’s condition is essential for effective medication recommendation.

Several studies have addressed sequential modeling. Yang et al [16] used dual medical sequences to represent medication history but did not account for intersequence relationships. Liu et al [17] developed 3 long short-term memory (LSTM) variants to model correlations across medical sequences but failed to consider the immediate influence of current clinical status. Although models like the Reverse Time Attention model (RETAIN) [18] use attention mechanisms to prioritize relevant historical visits and An et al [19] introduced hierarchical temporal modeling, these methods primarily focus on general temporal behavior without consideration for hypertension-specific patterns like long-term management or phased treatment adjustment.

Advanced designs have attempted to improve modeling capabilities. Le et al [20] used a memory-enhanced neural network to represent long-term dependencies, while Yang et al [21] adopted a residual mechanism to capture patient status transitions. Wu et al [22] introduced a transformer with a “copy-generate” mechanism to decide whether to reuse previous prescriptions. However, none of these methods fully addresses the task-specific temporal dynamics required for hypertension medication recommendations.

### Objective and Contributions

To address the aforementioned limitations, this study proposed CSRec, a novel hypertension medication recommendation framework that integrates the synergistic interactions of heterogeneous medical entities with selective modeling of temporal progression. CSRec is designed to capture cross-entity synergies through both heterogeneous and homogeneous graph construction based on EHR data, model time-aware patient representations using a temporal selectivity module that weighs current versus historical visits, and enhance safety by incorporating DDI information in the recommendation process.

The primary contributions of this paper are summarized in the following paragraphs.

We propose a novel medication recommendation model, named CSRec, specifically designed for hypertension treatment. CSRec effectively integrates the synergistic interactions and selective characteristics among diverse medical entities. By constructing a heterogeneous medical entity graph derived from EHRs, our model utilizes a graph attention mechanism to generate enhanced collaborative embeddings among medical entities. Additionally, a temporal selection mechanism was incorporated to simulate hypertension progression, thereby producing a comprehensive patient representation to facilitate accurate medication recommendations.

To better capture synergistic relationships between medical entities, we innovatively modified the traditional graph attention network (GAT) to focus more on neighboring node information, thus obtaining a more aggregated representation of the principal nodes.

Extensive experiments on the publicly available datasets Medical Information Mart for Intensive Care (MIMIC)-III [23] and MIMIC-IV [24] were conducted to validate the superiority and effectiveness of our proposed method.

## Methods

### Overview

**Figure 1.**
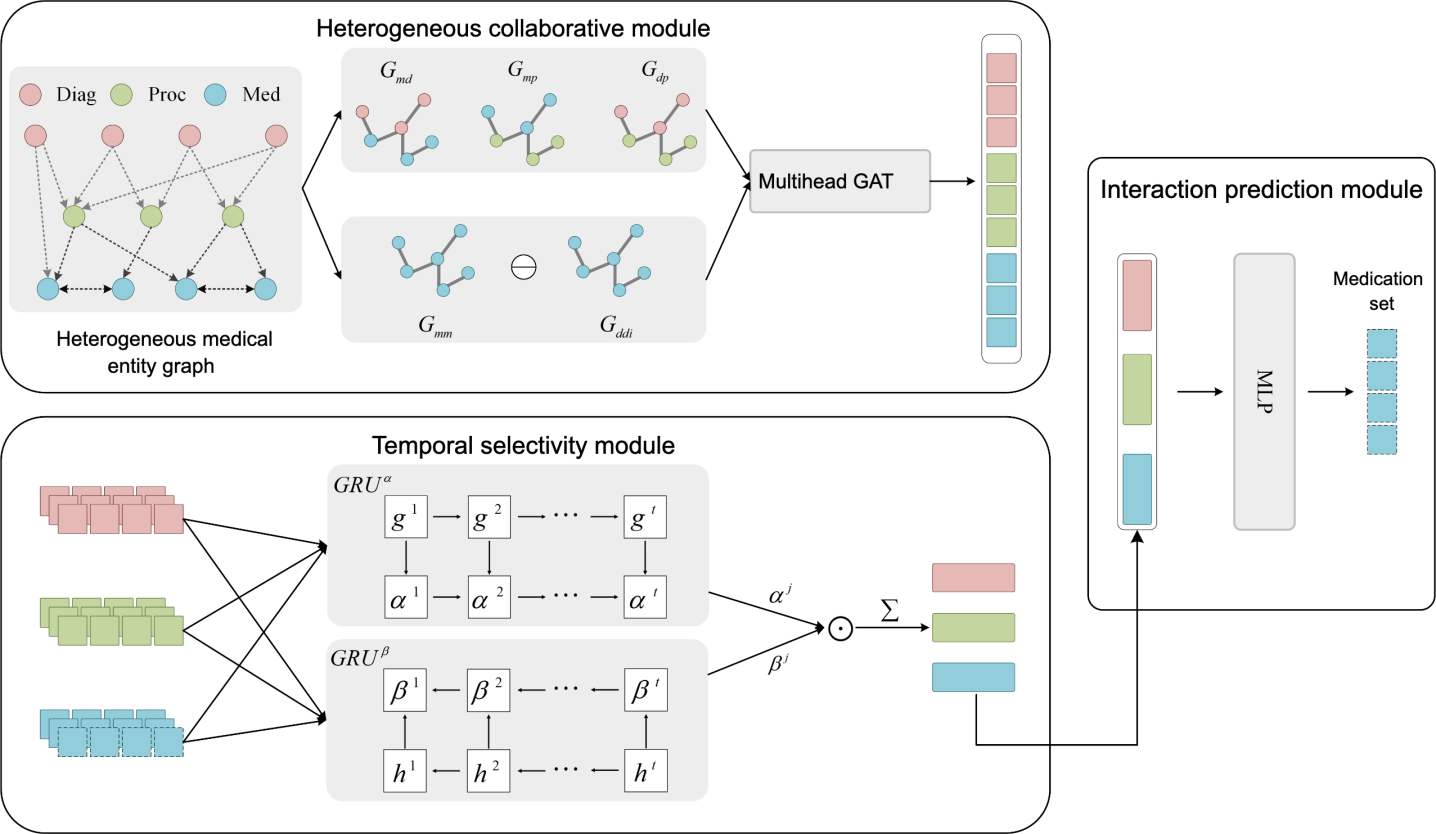
The framework of CSRec. Diag: diagnosis; GAT: graph attention network; GRU: gated recurrent unit; Med: medication; MLP: multilayer perceptron; Proc: procedures.

In this section, we present a comprehensive description of the CSRec model’s structure. As depicted in Figure 1, our model comprises 3 core modules for end-to-end hypertension medication recommendations: (1) The Heterogeneous Collaborative Module, with heterogeneous and homogeneous graph networks, learns entity-level representations by aggregating collaboration patterns between medical entities, which are then passed to the Temporal Selectivity Module; (2) the Temporal Selectivity Module, using a bidirectional selection mechanism, processes these entity-level representations to calculate relevance coefficients between current and previous visits, generating visit-level entity representations that are transmitted to the Interaction Prediction Module; and (3) the Interaction Prediction Module concatenates these visit-level entity representations to enrich entity information, forming a patient representation. This is converted into medication recommendation probabilities, with medications exceeding a threshold output as results.

### Problem Formulation

#### Electronic Health Records

EHRs encompass a variety of medical visit information collected from patients. For a specific patient, EHRs can be structured into a sequence comprising multiple clinical visit records, represented as $V=\left[V^{1},V^{2},\cdots ,V^{T}\right]$, where $V^{t}$ represents the t-th visit and T indicates the total number of visits for that patient. Specifically, each clinical visit $V^{t}$ can be represented as $V^{t}=\left[V_{d}^{t},V_{p}^{t},V_{m}^{t}\right]$, in which $V_{d}^{t}\in {\left\{0,1\right\}}^{\left|D\right|}$, $V_{p}^{t}\in {\left\{0,1\right\}}^{\left|P\right|}$, and $V_{m}^{t}\in {\left\{0,1\right\}}^{\left|M\right|}$ represent multihot encoded vectors corresponding to diagnosis, procedures, and medications, respectively. Here, the notation indicates the total number of distinct categories within each respective medical entity type.

#### Heterogeneous Medical Entities

In this paper, distinct medications were modeled as medication entities. Each medication recorded in the EHR corresponds to a unique medication entity, identified by a specific identifier denoted as $\left\{m_{1},m_{2},\cdots \right\}$, and each entity is independently embedded within the model using embeddings of identical dimensionality. Likewise, diagnoses and procedures were categorized into diagnostic entities and procedural entities, respectively. Collectively, these 3 categories of entities were termed heterogeneous medical entities.

#### Hypertension Medication Recommendation

Based on the patient’s current diagnostic information $V_{d}^{t}$, procedural data $V_{p}^{t}$, historical visit sequence $V=\left[V^{1},V^{2},\cdots ,V^{T-1}\right]$, and heterogeneous medical entity graph G, the model recommends appropriate antihypertensive medications ${\hat{y}}_{t}\in {\left\{0,1\right\}}^{\left|Μ\right|}$ to the patient.

### Heterogeneous Collaborative Module

For a specific patient, we first constructed a medication homogeneous graph, which was explicitly designed to characterize relationships between medications and provide a foundation for subsequent cross-entity modeling. This graph comprised 2 components, both of which take medications as the sole node type.

**Figure 2.**
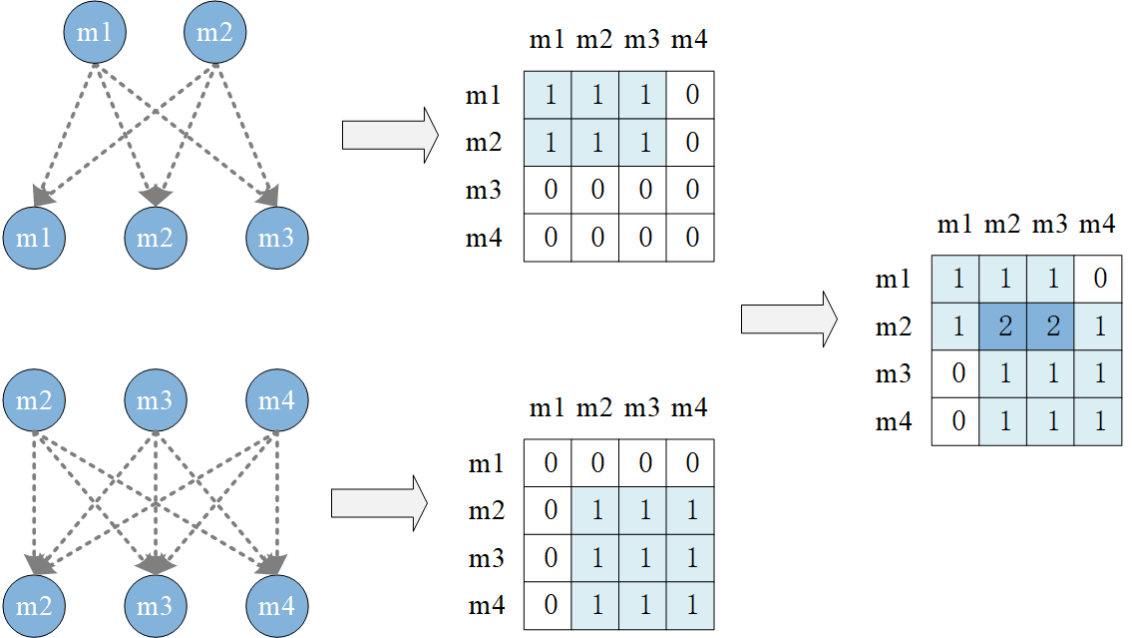
The medication-medication synergy matrix construction process.

One component is the medication collaboration graph $G_{mm}\left(t-1\right)=\left\{M\left(t-1\right),A^{mm}\right\}$, which serves to capture the patterns of combined medication use in clinical practice. Specifically, $M\left(t-1\right)$ denotes the collection of all prescribed medications from the patient’s previous visits, and $A^{mm}$ signifies the adjacency matrix representing collaborative interactions among medications. Each entry within this adjacency matrix indicates the initial collaboration weight between the respective medication nodes. The detailed procedure for generating this matrix is illustrated in Figure 2. Initially, $A^{mm}$ is set as a zero matrix. If medication i and medication j co-occur during a specific visit, then $A\left[i,j\right]=1$; if medication i and j co-occur across multiple visits, the corresponding value in $A^{mm}$ is incremented, with higher values indicating stronger collaboration between the medications.

Each medication node $m\in M\left(t-1\right)$ in the graph corresponds to its initial embedding vector, expressed as:

  (1) $$e_{m}=V_{m}^{t-1}E_{m}$$

Specifically, $E_{m}$ refers to the embedding matrix associated with medication entities. The selector vector $V_{m}^{t-1}$ specifically extracts the relevant embedding from $E_{m}$ for the medication at the previous time step t-1.

Moreover, some medications may have harmful interactions (DDI) and should be avoided when used together. To address this, we integrated the medication collaboration graph $G_{mm}$ with the medication safety graph $G_{ddi}$ , enhancing the comprehensive representation of the medication nodes. Notably, the methodology used to construct the medication safety graph $G_{ddi}$ closely mirrors that of $G_{mm}$. Specifically, we used $A^{ddi}$ as the adjacency matrix, where $A^{ddi}\left[i,j\right]=1$ signifies that the i-th and j-th medications exhibit a paired harmful medication interaction.

  (2) $$G_{m}=G_{mm}-\lambda G_{ddi}$$

Subsequently, we adopted a GAT-based graph neural approach to obtain embeddings for medication nodes within the medication graph $G_{m}$.

First, we utilized the attention mechanism to calculate the attention coefficients between a node and its neighbors, followed by normalization:

  (3) $$\alpha_{ij}^{\left(m\right)}=\frac{exp\left(g_{ij}^{\left(m\right)}\right)}{\sum_{k\in N_{i}^{\left(m\right)}}exp\left(g_{ik}^{\left(m\right)}\right)}$$

Here, $g_{ij}^{\left(m\right)}=LeakyℜLU\left(\beta^{{\left(m\right)}^{T}}\left(h_{i}^{\left(m\right)}∥h_{j}^{\left(m\right)}\right)\right)$ denotes the output of the LeakyReLU activation function applied to the linear transformation of the concatenated feature vectors $h_{i}^{\left(m\right)}$and $h_{j}^{\left(m\right)}$, parameterized by the learnable weight vector $\beta^{\left(m\right)}$. The notation $N_{i}^{\left(m\right)}$ represents the neighboring node set of node $m_{i}$ in the graph $G_{m}$, while ∥ signifies the vector concatenation operation.

Second, we used the calculated attention coefficients to perform weighted aggregation of neighbor nodes, thereby obtaining the representation of node $m_{i}$:

  (4) $$e_{i}^{\left(m\right)}=\sigma \left(\sum_{j\in N_{i}^{\left(m\right)}}\gamma_{ij}^{\left(m\right)}h_{j}^{\left(m\right)}\right)$$

To overcome the limitations posed by single-view attention, we further introduced a multihead attention mechanism, using a linear layer to map node representations into multiple subspaces, then aggregate representations under each subspace. Formally, this operation is expressed as:

  (5) $$e_{i}^{\left(m\right)}={∥}_{h=1}^{H}\sigma \left(\sum_{j\in N_{i}^{\left(m\right)\left(h\right)}}\gamma_{ij}^{\left(m\right)\left(h\right)}h_{j}^{\left(m\right)\left(h\right)}\right)$$

In this equation, H denotes the total number of attention heads, the superscript $\left(h\right)$ indicates the current attention head index, and ∥ represents concatenating outputs from different heads. Through this strategy, distinct node-specific information is captured across various dimensions and integrated from multiple subspaces, thereby significantly improving the precision and robustness of the learned node representations.

During the training process, each medication entity is updated according to the aforementioned steps, resulting in an aggregated representation of the medication set $V_{m}=\left\{v_{m}^{1},v_{m}^{2},\cdots v_{m}^{t-1}\right\}$.

Similarly, to capture the unique clinical relationships of hypertension, such as the interactions between comorbidities, long-term monitoring data, and combination therapy dynamics, we defined 3 heterogeneous complete graphs: diagnosis-procedure graph $G_{dp}\left(t\right)=\left\{D\left(t\right),P\left(t\right),A^{dp}\right\}$, medication-diagnosis graph $G_{md}\left(t\right)=\left\{M\left(t-1\right),D\left(t\right),A^{md}\right\}$, and medication-procedure graph $G_{mp}\left(t\right)=\left\{M\left(t-1\right),P\left(t\right),A^{mp}\right\}$. This established an association model that is crucial for accurate hypertension medication recommendations but has low correlation with other medication categories. Following a similar learning approach to the medication graph $G_{m}$, we first initialized the diagnosis and procedure nodes in the 3 heterogeneous graphs.

  (6) $$e_{d}=V_{d}^{t}E_{d},e_{p}=V_{p}^{t}E_{p}$$

Using the approach outlined in equations (3)-(5), we then learned from the diagnosis-procedure graph to obtain aggregated sets of diagnosis codes $V_{d}=\left\{v_{d}^{1},v_{d}^{2},\cdots v_{d}^{t}\right\}$ and procedure nodes $V_{p}=\left\{v_{p}^{1},v_{p}^{2},\cdots v_{p}^{t}\right\}$.

Finally, based on the aggregated medication nodes from the medication graph $G_{m}$ and the learned diagnosis and procedure nodes from the diagnosis-procedure graph $G_{dp}$, we learned from the medication-diagnosis graph $G_{md}$ and the medication-procedure graph $G_{mp}$, updating to obtain entity-level representations of the medication, diagnosis, and procedure sets:


$$V_{D}=\left\{V_{d}^{1},V_{d}^{2},\cdots V_{d}^{t}\right\}$$

$$V_{P}=\left\{V_{p}^{1},V_{p}^{2},\cdots V_{p}^{t}\right\}$$

(7) $$V_{M}=\left\{V_{m}^{1},V_{m}^{2},\cdots V_{m}^{t-1}\right\}$$

It is noteworthy that this study used a multihead GAT mechanism to learn and update nodes within the graph. Although traditional GAT models consider intrinsic features and aggregate neighboring features, our proposed heterogeneous graph-based method places greater emphasis on enhancing the embeddings of medications, diagnoses, and procedures by explicitly modeling interactions among diverse medical entities. Consequently, our model focuses more intensively on neighboring nodes to prevent excessive node information merging. To this end, we modified the standard GAT approach to explicitly focus on information derived from neighboring nodes within the heterogeneous graph learning process.

### Temporal Selectivity Module

To effectively model the temporal evolution of patient health conditions, this paper introduces a bidirectional temporal selection mechanism using gated recurrent units (GRUs), with 3 key innovations that distinguish it from conventional applications.

First, we adopted a bidirectional temporal architecture to capture multiscale temporal dependencies. Specifically, we first used $GRU^{\alpha }$ to learn the diagnostic sequence, generating forward diagnostic selection coefficients that emphasize the impact of historical visits on current states (eg, past hypertensive crisis records influencing present medication titration):

  (8) $$g^{1},g^{2},\cdots ,g^{t}=GRU_{d}^{\alpha }\left(V_{d}^{1},V_{d}^{2},\cdots ,V_{d}^{t}\right)$$

  (9) $$\alpha^{j}=tanh\left(W^{\alpha }g^{j}+b^{\alpha }\right),j=1,\cdots ,t$$

Concurrently, a backward $GRU^{\beta }$ was used in the reverse temporal order to learn the diagnostic sequence, generating backward selection coefficients at different time steps that highlight recent critical changes. For example, recent fluctuations in blood pressure require immediate therapeutic adjustment. This bidirectional design enhances computational stability while overcoming the limitations of unidirectional GRUs or traditional recurrent neural networks (RNNs), which often overlook either long-term or short-term temporal cues:

  (10) $$h^{t},h^{t-1},\cdots ,h^{1}=GRU_{d}^{\beta }\left(V_{d}^{t},V_{d}^{t-1},\cdots ,V_{d}^{1}\right)$$

  (11) $$\beta^{j}=tanhshrink\left(W^{\beta }h^{j}+b^{\beta }\right),j=t,\cdots ,1$$

Second, we proposed an adaptive selection coefficient integration strategy. Based on the generated bidirectional diagnostic selection coefficients, we can capture key visit information and entity information within the visit sequence, rather than relying on static aggregation methods. This allowed us to capture key visit information and entity interactions, thereby obtaining a diagnostic representation that integrates historical context with current needs:

  (12) $$d^{t}=\sum_{j=1}^{t}\alpha^{j}\beta^{j}⊙V_{d}^{j}$$

Third, leveraging GRU’s inherent advantages, our design achieved computational efficiency without sacrificing performance. Compared with traditional RNNs, GRU effectively mitigates gradient vanishing issues; relative to LSTMs, its simplified gating mechanism reduces parameter complexity by avoiding redundant memory cells, resulting in faster training efficiency, a critical advantage for handling large-scale longitudinal EHR data in medication recommendation tasks. After a series of similar processing steps, we obtained the patient’s final procedural and medication representations.

  (13) $$p^{t}=\sum_{j=1}^{t}\alpha^{j}\beta^{j}⊙V_{p}^{j},m^{t-1}=\sum_{j=1}^{t-1}\alpha^{j}\beta^{j}⊙V_{m}^{j}$$

### Interaction Prediction Module

Based upon the outputs generated by the aforementioned modules, for the patient’s t-th visit, we concatenated the medical entity sequence to make medication recommendations:

  (14) $$y^{t}=\sigma \left(\left[d^{t};p^{t};m^{t-1}\right]\right)$$

In our approach, the medication recommendation task is formulated as a multilabel classification problem [25,26]. To address the complexities and potential imbalances in medical datasets, we used a comprehensive strategy during model training. We enhanced the model’s generalization capabilities and mitigated overfitting through the use of regularization techniques, which limit the complexity of the parameters learned. Additionally, an early-stopping mechanism was implemented to curtail training based on validation set performance. To optimize parameters, we used the Adam optimization algorithm [27], which minimizes the binary cross-entropy loss function to promote efficient and stable model convergence.

  (15) $$L=-\sum_{t=1}^{T}\sum_{i=1}^{|M|}y_{i}^{t}\log{\hat{y}}_{i}^{t}+\left(1-y_{i}^{t}\right)\log\left(1-{\hat{y}}_{i}^{t}\right)$$

### Ethical Considerations

This study made use of the standardized, publicly available MIMIC-III and MIMIC-IV datasets from the Massachusetts Institute of Technology [23,24] and was therefore deemed exempt from ethical approval requirements. Prior to their release, these datasets underwent comprehensive ethical review and privacy protection processes conducted by the data provider (Massachusetts Institute of Technology). These processes included deidentifying all personally identifiable information in patients’ EHRs, such as names, hospital admission numbers, and dates of birth. Additionally, the datasets’ usage license explicitly covers secondary analysis scenarios for academic research, eliminating the need for users to obtain additional ethical approval independently. Since this study did not involve independent collection of human subject data and solely relied on the aforementioned publicly available and compliant existing datasets for secondary analysis, we do not possess separate ethical approval documents to provide. The ethical approval statement is published on the official website of the data provider [23,24], and we can be contacted to obtain the original copy of the datasets’ usage license.

## Results

### Dataset Description

Our experiments were conducted using the MIMIC-III v1.4 and MIMIC-IV v2.2 datasets provided by the Massachusetts Institute of Technology. The datasets comprise medical records collected from patients admitted to intensive care units, such as diagnoses, procedures, and medications. The diagnostic and procedural information uses the *International Classification of Diseases, Ninth Revision* (*ICD-9*) coding system. To study medication recommendations for patients with hypertension, this paper extracted relevant data from the aforementioned datasets based on the *ICD-9* hypertension codes under the guidance of clinical experts. Meanwhile, referring to previous research norms [4,21,22], patients who completed at least two visits were included, and medications with a frequency <2000 occurrences were excluded.

During data analysis, we observed that the number of frequently used antihypertensive medications was relatively limited and the recommendations based on overly broad Anatomical Therapeutic Chemical (ATC) categories often lacked the specificity needed for precise clinical decision-making. Given the systematic medication classification framework of the ATC classification system, this study overcame the limitation of previous studies that only focused on the ATC04 level for recommendation. It not only predicted the medication categories at the ATC04 level (such as C02A, anti-adrenergic medications with central effects) but also further refined to the specific medication types at the ATC05 level (such as C02AA, reserpine-like medications).

After data extraction, we carried out meticulous preprocessing. We normalized features to standardize the scale of different variables, which prevented certain features from dominating others during model training. Moreover, we selectively chose variables most relevant to hypertension medications. Finally, the preprocessed dataset was partitioned into training, validation, and testing subsets according to a ratio of $\frac{2}{3}:\frac{1}{6}:\frac{1}{6}$.

Detailed information about the dataset used in the experiments and examples from patients are shown in Table 1 and Table 2.

**Table 1.** Detailed information for the experimental datasets, by Anatomical Therapeutic Chemical (ATC)–level encoding.

Item	MIMIC-III^a^		MIMIC-IV	
	ATC04	ATC05	ATC04	ATC05
Patients, n	3115	3109	19,609	19,609
Visits, n	7308	7263	55,239	55,236
Diagnoses, n	1966	1965	2000	2000
Procedures, n	1145	1145	5488	5488
Medications, n	14	18	14	18
Number of visits, mean	2.3460	2.3361	2.8169	2.8169
Number of diagnoses, mean	10.8927	10.9262	9.3666	9.3666
Number of procedures, mean	4.0463	4.0609	2.5492	2.5492
Number of medications, mean	1.5508	1.4400	1.0804	1.1352

a MIMIC: Medical Information Mart for Intensive Care.

**Table 2.** Samples from electronic health records.

Sub_ID	Hadm_ID	Diagnoses (Anatomical Therapeutic Chemical code)	Procedures	Medications
10001217	24597018	3240, 3484, 3485, 5180, 340, 04109, 3051, 4019, V168,V161	139, 331, 3897	HydrALAzine, LeVETiracetam, Vancomycin ,Bisacodyl, Meropenem,…
10001217	27703517	3240, 3485, 340, 04102, 04184, 4019, 3051	139	HydrALAzine, Vancomycin, Meropenem, Bisacodyl, Lidocaine,…

### Evaluation Metrics

To validate the effectiveness of CSRec, the evaluation metrics described in the following sections were used.

#### Jaccard Similarity Coefficient

A higher Jaccard coefficient reflects greater overlap between the predicted medication set and the actual medication set.

  (16) $$Jaccard=\frac{1}{T}\sum_{t=1}^{T}\frac{\left|y^{t}\cap {\hat{y}}^{t}\right|}{\left|y^{t}\cup {\hat{y}}^{t}\right|}$$

#### Area Under the Curve of Precision-Recall

A high area under the curve of precision-recall (PRAUC) indicates that the model recommends appropriate medications while keeping a low error rate.

  (17) $$\Delta ℜcall(i{)}^{t}=ℜcall(i{)}^{t}-ℜcall(i-1{)}^{t}$$

  (18) $$PRAUC=\frac{1}{T}\sum_{t=1}^{T}\sum_{i=1}^{\left|M\right|}Precision{\left(i\right)}^{t}\Delta ℜcall{\left(i\right)}^{t}$$

#### *F*_1_-Score

The elevated *F*_1_-score metric in medication recommendations signifies an optimal equilibrium between minimizing false negatives and maintaining classification specificity.

  (19) $$Precision^{t}=\frac{\left|y^{t}\cap {\hat{y}}^{t}\right|}{\left|{\hat{y}}^{t}\right|},\;Recall^{t}=\frac{\left|y^{t}\cap {\hat{y}}^{t}\right|}{\left|y^{t}\right|}$$

  (20) $$F1=\frac{1}{T}\sum_{t=1}^{T}\frac{2\times Precision^{t}\times ℜcall^{t}}{Precision^{t}+ℜcall^{t}}$$

#### DDI Rate

A lower DDI rate ensures that the recommended combination of medications is safer in clinical practice.


(21) $$DDI=\frac{1}{T}\sum_{t=1}^{T}\frac{\sum_{i=1}^{|{\hat{y}}^{t}|}\sum_{j=i+1}^{|{\hat{y}}^{t}|}1\{A_{d}[{\hat{y}}_{i}^{t},{\hat{y}}_{j}^{t}]\}}{\sum_{i=1}^{|{\hat{y}}^{t}|}\sum_{j=i+1}^{|{\hat{y}}^{j}|}1}$$

### Comparative Models

The following baseline models were selected for comparison with the proposed CSRec:

- Logistic regression (LR) uses L2 regularization.
- Ensemble of classifier chains (ECC) [28] enhances predictions by connecting multiple classifiers, typically applied to multilabel classification scenarios for performance optimization.
- RETAIN [18] implements bidirectional temporal attention architecture designed for sequential clinical prediction tasks like treatment recommendation.
- Learn to Prescribe (LEAP) [29] uses RNNs to extract meaningful representations during current medical visits and generate medication sequences.
- Dual memory neural computer (DMNC) [20] incorporates dual-memory neural components to model asynchronous therapeutic pattern interactions.
- GAMENet [11] adopts graph-based memory architecture combining medication interaction knowledge and querying longitudinal EHR data for medication retrieval.
- MICRON [21] analyzes EHR temporal dynamics to adaptively optimize medication combinations upon symptom evolution.
- SafeDrug [16] evaluates molecular-level medication-patient compatibility to suggest safer therapeutic regimens.
- COGNet [22] implements medication copy or predict strategy integrating historical effective prescriptions into current recommendations.
- MoleRec [14] predicts medication mechanisms and interactions via molecular-patient relationships for personalized, safer recommendations.
- CausalMed [9] identifies medical entity causal relationships via causal discovery, accounting for dynamic health condition differences to generate causally linked recommendations.

### Performance Comparison

In this section, we conducted an extensive comparative analysis between CSRec and the previously described baseline medication recommendation models to evaluate its effectiveness. Experimental results on the MIMIC-III v1.4 and MIMIC-IV 2.2 datasets are shown in Table 3 (“↑” indicates a preference for larger values, “↓” indicates a preference for smaller values).

**Table 3.** The comparison of experimental results on datasets of Anatomical Therapeutic Chemical (ATC)04 and ATC05 codes.

Model	Jaccard similarity coefficient↑^a^		PRAUC↑^b^		*F*_1_-score↑		DDI↓^c^^,^^d^	
	ATC04	ATC05	ATC04	ATC05	ATC04	ATC05	ATC04	ATC05
MIMIC-III^e^								
LR^f^	0.5203	0.4919	0.8117	0.7726	0.6347	0.5972	0.2951	0.3569
ECC^g^	0.5438	0.5056	0.8278	0.7846	0.6593	0.6127	0.3012	0.3979
RETAIN^h^	0.5540	0.5210	0.8143	0.7874	0.6783	0.6449	0.3921	0.5068
LEAP^i^	0.5228	0.5082	0.6346	0.6162	0.6405	0.6217	0.2627	0.3648
DMNC^j^	0.5342	0.5097	0.8225	0.7696	0.6463	0.6277	0.2365	0.2615
GAMENet^k^	0.5497	0.5169	0.8239	0.7861	0.6710	0.6308	0.2366	0.4199
MICRON	0.5352	0.5146	0.8285	0.7884	0.6423	0.6145	0.2357	0.2658
SafeDrug	0.5474	0.5063	0.8252	0.7764	0.6484	0.6146	0.2329^l^	0.1975^l^
COGNet	0.5325	0.5075	0.7972	0.7762	0.6448	0.6167	0.2902	0.3587
MoleRec	0.5501	0.5189	0.8261	0.7825	0.6716	0.6299	0.2709	0.3491
CausalMed	0.5626	0.5340	0.8226	0.7877	0.6741	0.6394	0.2340	0.2679
CSRec	0.5801^l^	0.5534^l^	0.8356^l^	0.8123^l^	0.6895^l^	0.6602^l^	0.2351	0.2663
MIMIC-IV								
LR	0.4248	0.3518	0.7426	0.6873	0.5125	0.4315	0.2821	0.2734
ECC	0.4389	0.3779	0.7498	0.6923	0.5305	0.4624	0.3064	0.3047
RETAIN	0.5349	0.5045	0.7964	0.7641	0.6356	0.6008	0.3431	0.3197
LEAP	0.4186	0.3884	0.5047	0.4683	0.5326	0.4932	0.0104^l^	0.0506^l^
DMNC	0.5139	0.4787	0.7678	0.7277	0.5392	0.5094	0.2801	0.2792
GAMENet	0.5151	0.4738	0.7781	0.7326	0.6069	0.5662	0.2884	0.3050
MICRON	0.4297	0.3716	0.7543	0.7056	0.5151	0.4431	0.2866	0.2557
SafeDrug	0.5173	0.4767	0.7770	0.7313	0.6109	0.5648	0.3084	0.3255
COGNet	0.5087	0.4568	0.7448	0.7009	0.5948	0.5502	0.3261	0.3835
MoleRec	0.5275	0.4838	0.7624	0.7267	0.6262	0.5801	0.2733	0.2993
CausalMed	0.5440	0.5225	0.7812	0.7509	0.6412	0.6099	0.2677	0.2855
CSRec	0.5582^l^	0.5307^l^	0.8069^l^	0.7655^l^	0.6483^l^	0.6195^l^	0.2760	0.2931

a ↑ indicates a preference for larger values.

b PRAUC: area under the curve of precision-recall.

c DDI: drug-drug interaction.

d ↓ indicates a preference for smaller values.

e MIMIC: Medical Information Mart for Intensive Care.

f LR: logistic regression.

g ECC: ensemble of classifier chains.

h RETAIN: Reverse Time Attention model.

i LEAP: Learn to Prescribe.

j DMNC: dual memory neural computer.

k GAMENet: Graph-Augmented Memory Networks.

l Optimal data.

After detailed analysis, we observed several key findings, as described in the following paragraphs.

The CSRec model proposed in this paper outperformed most comparative models across multiple evaluation metrics, fully demonstrating its significant effectiveness in hypertension medication recommendation.

LR and ECC performed poorly, primarily because these methods only focus on the patient’s current clinical condition, neglecting the influence of historical medical data on present treatment decisions. In contrast, the model presented in this paper, along with RETAIN, GAMENet, and other models integrating longitudinal medical histories, performed better, highlighting the importance of capturing historical medical information in hypertension medication recommendations.

In comparison with longitudinal models such as RETAIN, GAMENet, and COGNet, CSRec maintained superior performance, with a particularly notable enhancement in Jaccard scores. This superiority can be attributed to 2 core architectural advancements. The Temporal Selectivity Module enables accurate simulation of patient disease progression by assigning enhanced weights to clinically critical historical information, thereby ensuring that recommendations adhere to consistent clinical reasoning frameworks. Meanwhile, the Heterogeneous Collaborative Module facilitates effective capture of synergistic relationships among heterogeneous medical entities (diagnoses, procedures, medications), enabling the identification of medication combinations that are clinically coherent and reflective of real-world coprescription patterns. Collectively, these innovations augment the degree of overlap between recommended and actually prescribed medications, as empirically validated by the elevated Jaccard index.

Although the CSRec model introduced in this study does not achieve the highest score on the DDI metric, it still ranks as suboptimal. This is mainly because the best performing LEAP model only considers the patient’s present health status, resulting in the recommendation of fewer medications and consequently yielding the lowest DDI scores.

### Ablation Study

To systematically assess the contribution and validity of each module within CSRec, ablation experiments were conducted on MIMIC-IV by comparing CSRec with its variants:

- WO_S removes the selection module based on the cyclic mechanism.
- WO_C removes the collaborative module based on the heterogeneous graph.
- GAT_GCN replaces the multihead GAT in the collaborative module with a graph convolutional network (GCN).

**Figure 3.**
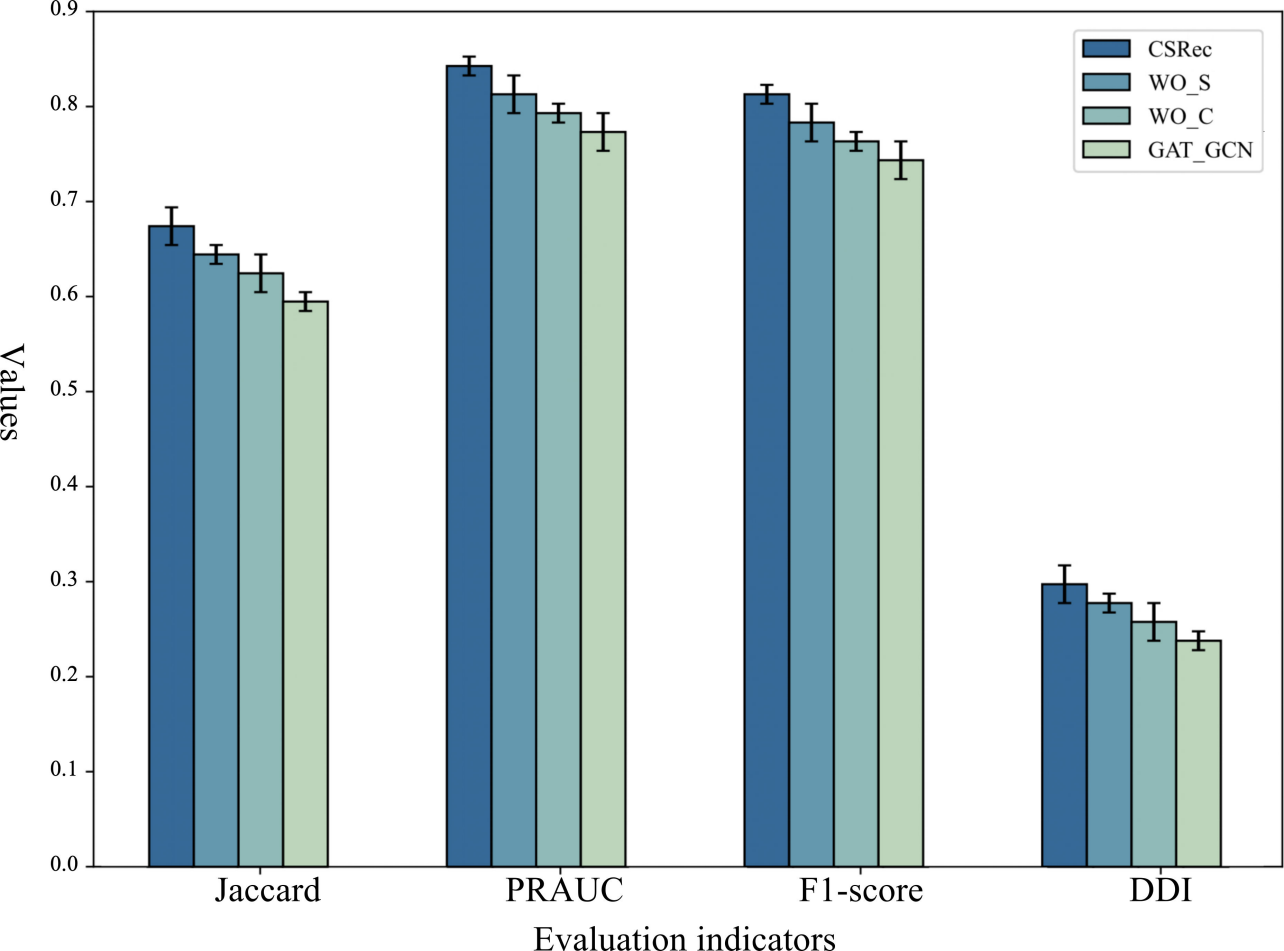
The ablation study of different components. DDI: drug-drug interaction; GAT_GCN: variant replacing the multihead graph attention network in the collaborative module with a graph convolutional network; Jaccard: Jaccard similarity coefficient; PRAUC: area under the curve of precision-recall; WO_C: variant removing the collaborative module based on the heterogeneous graph; WO_S: variant removing the selection module based on the cyclic mechanism.

The results displayed in Figure 3 indicate that the performance of CSRec declined when any module is removed or replaced, demonstrating that each component of CSRec is indispensable. The observed performance drops in evaluation metrics (Jaccard, PRAUC, *F*_1_-score, and DDI) can be attributed to the unique functional roles of each module: The collaborative module effectively modeled the correlations between different medical events during each visit, and its removal (WO_C) led to incomplete capture of event associations, resulting in a decrease in recommendation accuracy as medical entity representations lost contextual relevance. The selective module globally modeled the patient’s historical medical data, and its absence (WO_S) caused the model to fail at emphasizing critical time points in disease progression, leading to a decline in metrics as the temporal continuity of hypertension development was disrupted. When replacing multihead GAT with GCN (GAT_GCN), the loss of the attention mechanism resulted in insufficient differentiation of important medical entity relationships, reducing recommendation precision compared with the original model as convolutional operations cannot dynamically weight heterogeneous graph information.

Additionally, this study further explored the impact of different types of collaborative medical entity information (diagnosis-medication, procedure-medication, diagnosis-procedure, and medication-medication) on hypertension medication recommendations. The variants were designed as follows:

- WO_DM removes diagnosis-medication interaction information.
- WO_PM removes procedure-medication interaction information.
- WO_DP removes diagnosis-procedure interaction information.
- WO_MM removes medication co-occurrence and interaction information.

**Figure 4.**
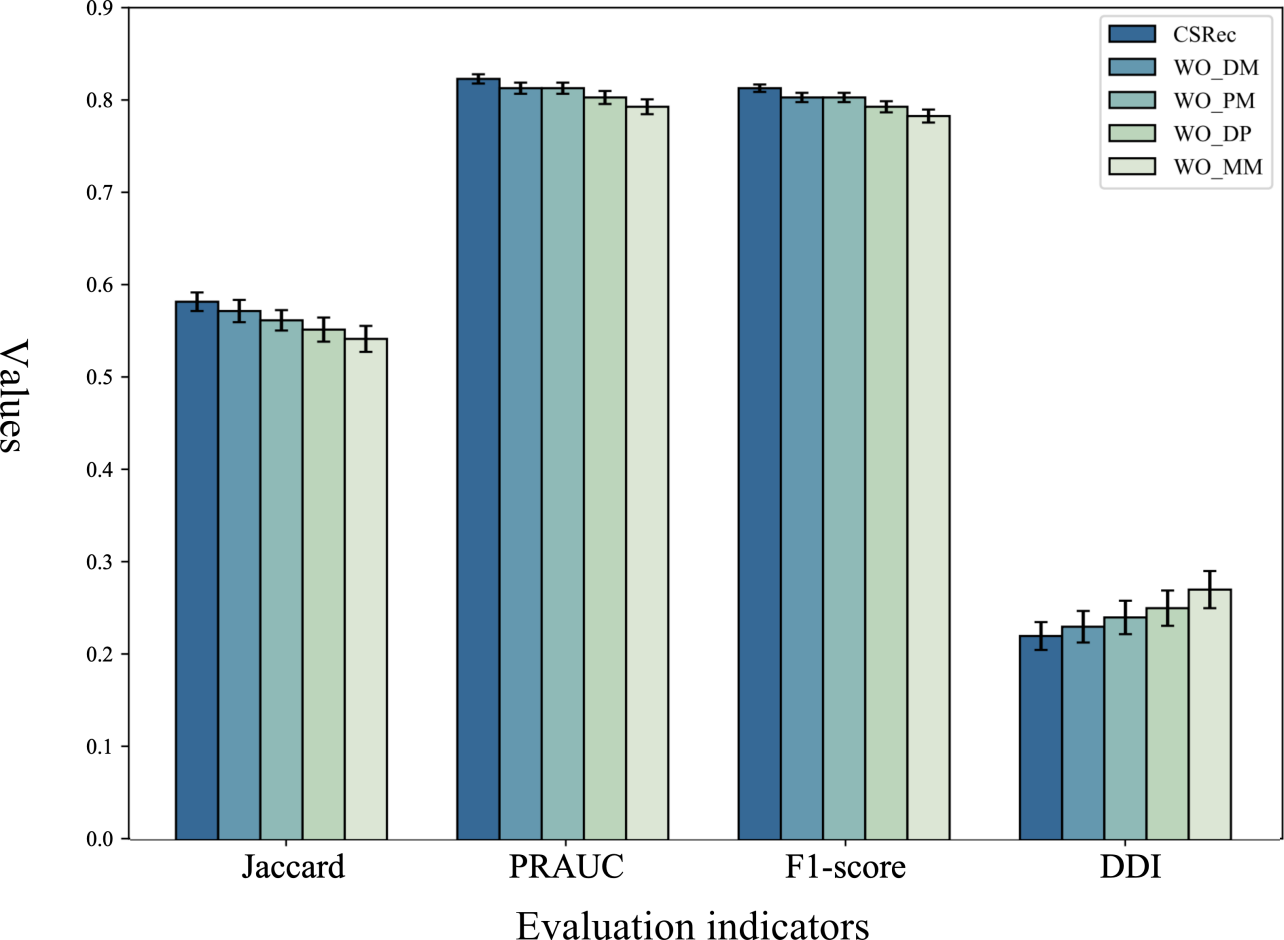
The ablation study of different medical entities. DDI: drug-drug interaction; Jaccard: Jaccard similarity coefficient; PRAUC: area under the curve of precision-recall; WO_DM: variant removing diagnosis-medication interaction information; WO_DP: variant removing diagnosis-procedure interaction information; WO_MM: variant removing medication co-occurrence and interaction information; WO_PM: variant removing procedure-medication interaction information.

As illustrated in Figure 4, CSRec’s performance decreased when any single type of collaborative medical entity information (WO_DM, WO_PM, WO_DP) was removed, confirming the strong correlation between such information and the medication recommendation task. The specific decline in metric degree corresponded to the clinical relevance of each information type: Removing diagnosis-medication interactions (WO_DM) reduced metrics, as diagnostic information directly guides first-line hypertension medication selection. The WO_PM group’s drop stemmed from lost procedure-related medication adjustments (eg, postinterventional anticoagulation needs). For WO_DP, the decline reflects disrupted diagnosis-procedure logical chains that inform therapeutic medication choices.

Notably, removing medication co-occurrence and interaction information (WO_MM) caused a significant decline in the DDI risk rate of the medication recommendation performance. This substantial change is explained by the frequent use of combination therapy in hypertension management. Without medication interaction data, the model cannot avoid contraindicated medication pairs, while the loss of co-occurrence patterns reduces the accuracy of synergistic medication recommendations. This underscores the essential role of medication co-occurrence and DDI information for ensuring hypertension treatment efficacy and safety.

### Parameter Sensitivity

**Figure 5.**
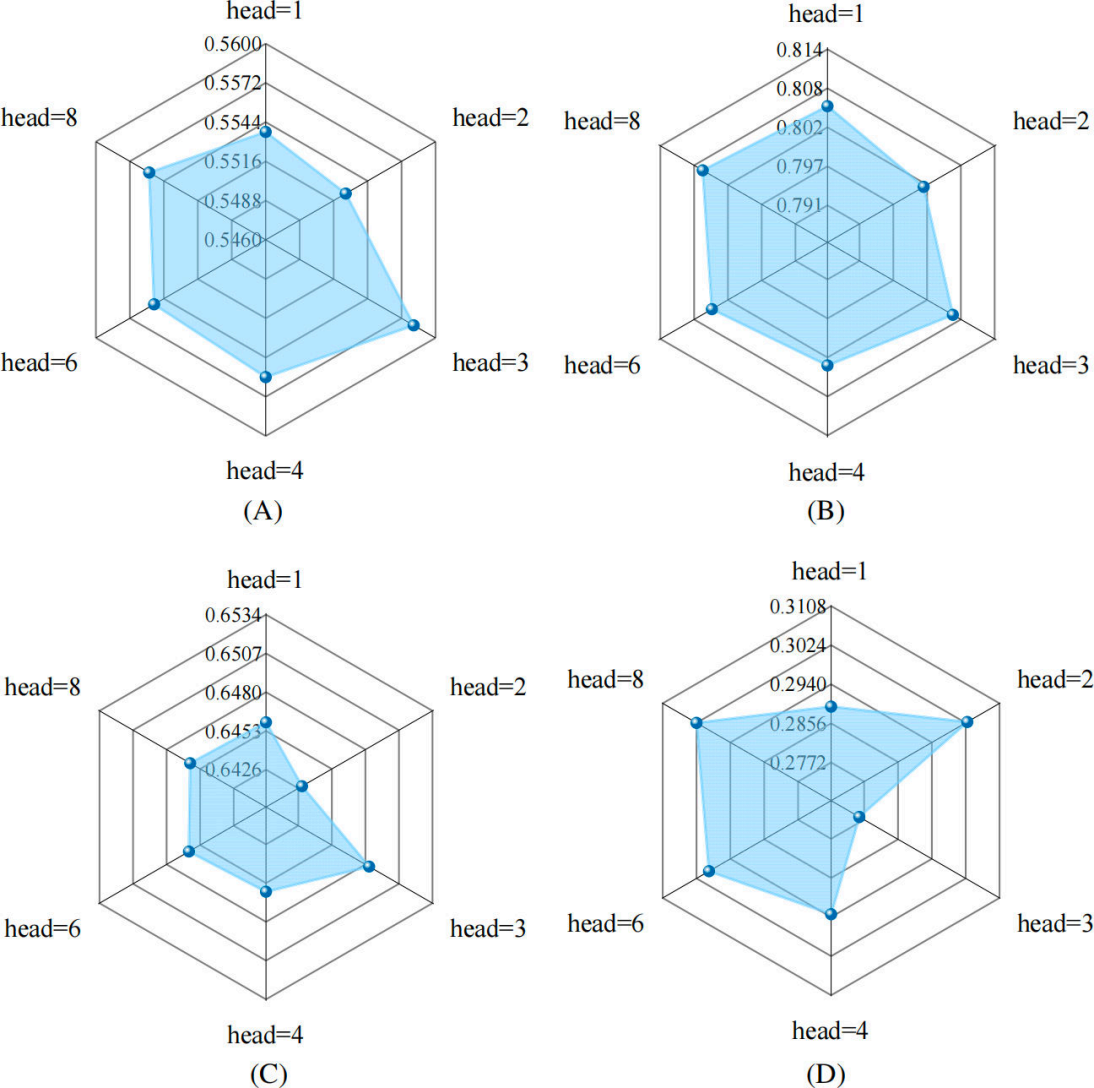
The effect of different numbers of heads in the multihead graph attention network (GAT), as measured using (A) the Jaccard similarity coefficient, (B) area under the curve of precision-recall (PRAUC), (C) *F*_1_-score, and (D) drug-drug interaction (DDI) rate.

In order to investigate how varying the number of attention heads in the multihead graph attention mechanism within the heterogeneous collaborative module impacted model efficacy, multiple experiments were conducted on the MIMIC-IV dataset. The outcomes of these experiments, comparing different head counts, are presented in Figure 5.

A setting of 3 attention heads (head=3) achieved optimal performance. Consequently, this configuration (head=3) was adopted consistently throughout the remaining experiments in this study. Moreover, the experimental results illustrated that variations in the number of attention heads led to minimal performance differences, highlighting the stability and reliability of the proposed model.

Furthermore, to investigate the impact of the number of included hypertension medications on model performance, we conducted a sensitivity analysis by varying the minimum occurrence threshold for medication inclusion from 500 to 3000. The number of medications ranged from 26 (≥500) to 9 (≥3000).

As shown in Table 4, performance across all 4 metrics—Jaccard, PRAUC, *F*_1_-score, and DDI—remained remarkably stable, with only marginal fluctuations. Notably, the best performance was observed at the 2000 threshold, which is used in our paper. This finding supported our design choice and indicated that our framework remained robust even under different medication inclusion criteria, alleviating concerns regarding limited sample size. In addition, setting a lower threshold introduced lower-frequency medications that may lack sufficient clinical representation, potentially compromising the statistical reliability of training. Thus, the current configuration struck a reasonable trade-off between medication variety and prediction stability.

**Table 4.** Sensitivity analysis of the medication frequency threshold.

Minimum medication occurrence	Medications, n	Jaccard similarity coefficient↑^a^	PRAUC↑^b^	*F*_1_-score↑	DDI↓^c^^,^^d^
≥500	26	0.5531	0.7983	0.6428	0.2847
≥1000	20	0.5554	0.8027	0.6456	0.2802
≥1500	17	0.5576	0.8049	0.6471	0.2781
≥2000	14	0.5582^e^	0.8069^e^	0.6483^e^	0.2760^e^
≥2500	12	0.5568	0.8051	0.6475	0.2766
≥3000	9	0.5560	0.8050	0.6452	0.2764

a ↑ indicates a preference for larger values.

b PRAUC: area under the curve of precision-recall.

c DDI: drug-drug interaction.

d ↓ indicates a preference for smaller values.

e Optimal data.

### Case Study

**Figure 6.**
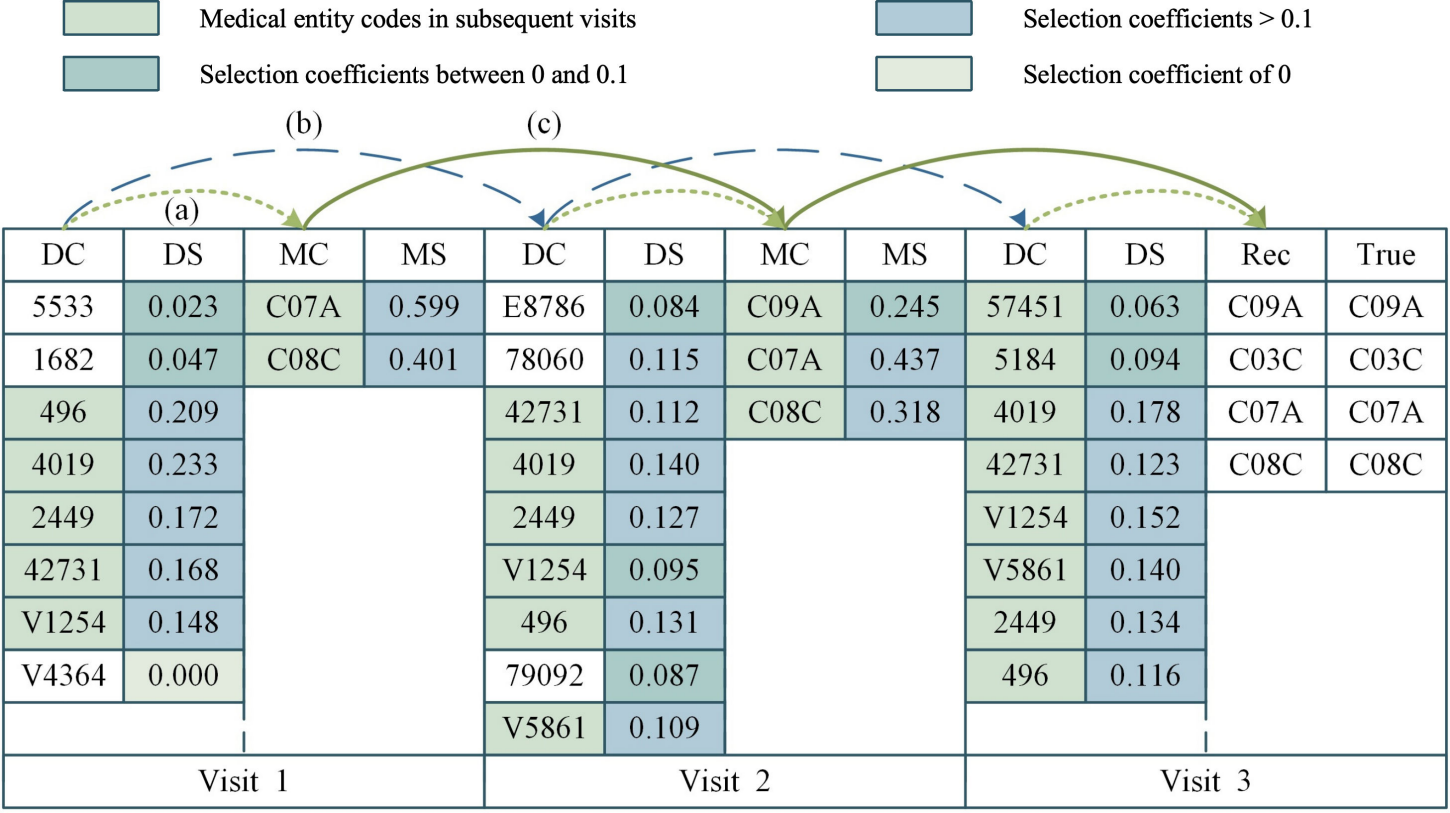
Case study. DC: diagnosis code; DS: diagnosis selection coefficient; MC: medication code; MS: medication selection coefficient; Rec: recommended medication; True: prescribed medication.

To visualize and validate our core innovations, which included heterogeneous collaborative interaction modeling and the temporal selectivity mechanism, we randomly selected a patient from the test set of the MIMIC-IV dataset for a case study. Taking diagnosis and medication entities as examples, the entire learning process is shown in Figure 6.

Initially, in the collaborative process (a), the model’s collaborative interaction mechanism was showcased: In the visit-specific level, the model dynamically enhanced the integration of diagnostic information into medication recommendation reasoning by assigning context-aware interaction weights to the interplays between diagnostic codes (DC column) and medication codes (MC column). For the first visit of the illustrative patient, the DC column encompasses comorbidity-related codes, including chronic obstructive pulmonary disease (*ICD-9*: 496), hypothyroidism (*ICD-9*: 2449), atrial fibrillation (*ICD-9*: 42731), and a documented history of cardiovascular disease (*ICD-9*: V1254), while the MC column includes hypertension-targeted agents such as selective β1-receptor blockers (ATC-04: C07A) and dihydropyridine calcium channel blockers (ATC-04: C08C). This aligns with our innovation of capturing disease-specific collaborative patterns, evident in the consistently higher selection coefficients for hypertension-related diagnostic codes in each DC column, which prioritizes the disease context critical for hypertension treatment.

Furthermore, to demonstrate our temporal selectivity mechanism, the case study highlights how the model captures longitudinal EHR dependencies. Given hypertension’s chronic nature, recommended medications depend on both current (third visit) diagnoses (DC column) and historical data. As shown in step (b), diagnostic codes from previous visits received higher selection coefficients, while step (c) reveals that historical medication codes were similarly prioritized. This reflects our model’s ability to dynamically weight temporal information, addressing the evolving nature of hypertension and representing an advantage over static models.

## Discussion

This paper presents the CSRec model for hypertension medication recommendation, which is based on the collaboration and selection of heterogeneous medical entities. By effectively capturing relationships among diverse medical entities and combining the temporal evolution characteristics of clinical entities, the model provides more precise and effective medication guidance for hypertension treatment. Future research will focus on 3 main areas: (1) in-depth exploration of the multilabel imbalance problem existing in the current method, (2) further investigation of the model’s performance in addressing the cold-start problem, and (3) exploration of recommendations regarding medication dosages and medication types.

## Abbreviations

ATC: Anatomical Therapeutic Chemical
CompNet: competitive neural network
DDI: drug-drug interaction
DMNC: dual memory neural computer
ECC: ensemble of classifier chains
EHR: electronic health record
G-BERT: graph-augmented bidirectional encoder representations from transformers
GAMENet: Graph-Augmented Memory Networks
GAT: graph attention network
GCN: graph convolutional network
GCT: graph convolutional transformer
GRU: gated recurrent unit
GT-BEHRT: graph transformers of bidirectional encoder representations from transformers on EHRs
HeteroMed: Heterogeneous Information Network for Medical Diagnosis
*ICD-9*: *International Classification of Diseases, Ninth Revision*
LEAP: Learn to Prescribe
LR: logistic regression
LSTM: long short-term memory
MiME: Multilevel Medical Embedding
MIMIC: Medical Information Mart for Intensive Care
PRAUC: area under the curve of precision-recall
RETAIN: Reverse Time Attention model
RNN: recurrent neural network
